# Supplementary material for: Repression of RNA polymerase by the archaeo-viral regulator ORF145/RIP
Source: Nat Commun. 2016 Nov 24;7:13595. doi: 10.1038/ncomms13595 (PMC5123050; doi:10.1038/ncomms13595)
Supplement: Supplementary Information — Supplementary Figures 1-5 and Supplementary Table 1 and 2, Supplementary Reference. [file ncomms13595-s1.pdf]

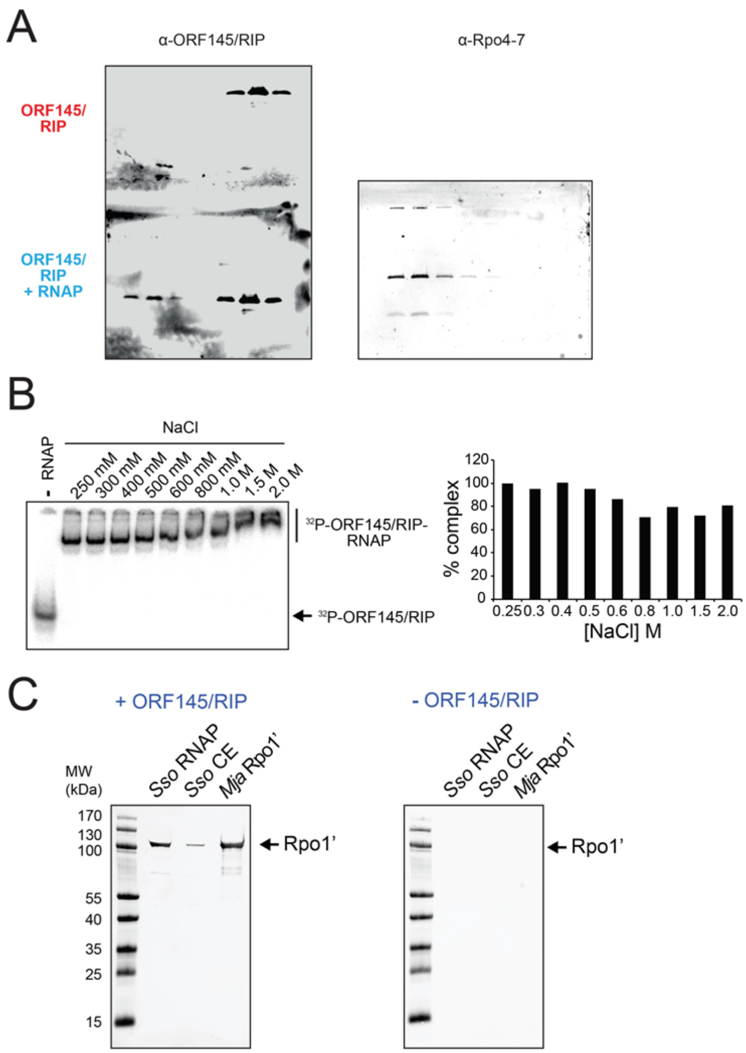


**Supplementary Figure 1: ORF145/RIP–RNAP binding.**

(A) Uncropped versions of the Western blots shown in Figure 1A. (B) EMSA analysis of $^{32}P$-ORF145/RIP-RNAP complexes formed at a range of salt concentrations showed that the ORF145/RIP-RNAP complex is stable at high (2 M) NaCl concentrations. (C) Western blot performed in the absence of ORF145/RIP protein showing that anti-ORF145/RIP does not display any non-specific binding to components in the Sso cell extract, the purified Sso RNAP or the recombinant Mja Rpo1′ protein.

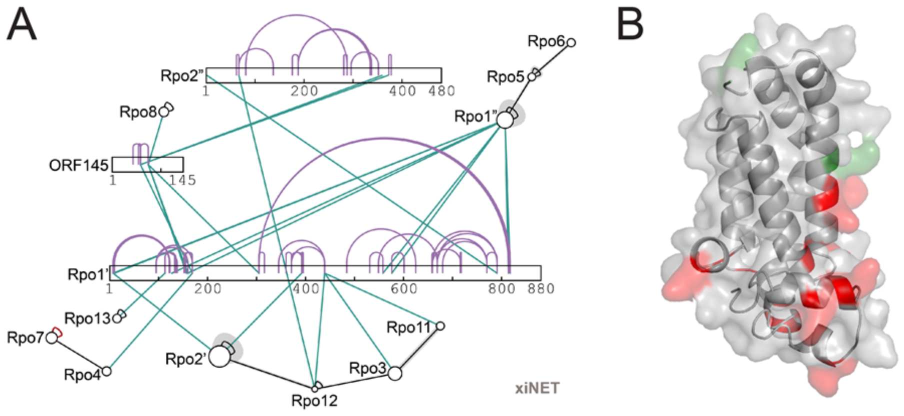


**Supplementary Figure 2: ORF145/RIP–RNAP cross-linking network.**

(A) Complete cross-linking network between RNAP subunits and ORF145/RIP using XiNet [1]. (B) Homology model of ORF145/RIP highlighting the two cross-linked lysine residues K58 and K74 in green, and the unreactive lysine residues in red.

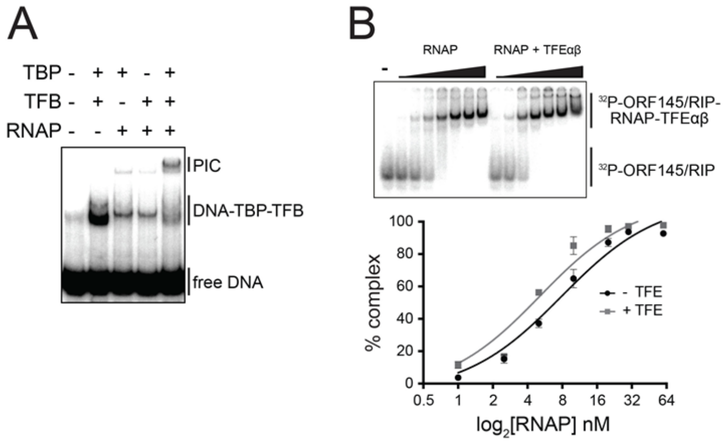


**Supplementary Figure 3: PIC formation in EMSAs and the effect of TFE $\alpha$ $\beta$ on ORF145/RIP-RNAP affinity.**

(A) EMSA showing the dependency of PIC formation on TBP and TFB. The PIC only forms in the presence of TBP, TFB and RNAP (lane 5). (B) EMSA of ORF145/RIP-RNAP binding -/+ TFEαβ using radiolabeled ORF145/RIP. The signals were quantified and plotted using the tight binding equation.

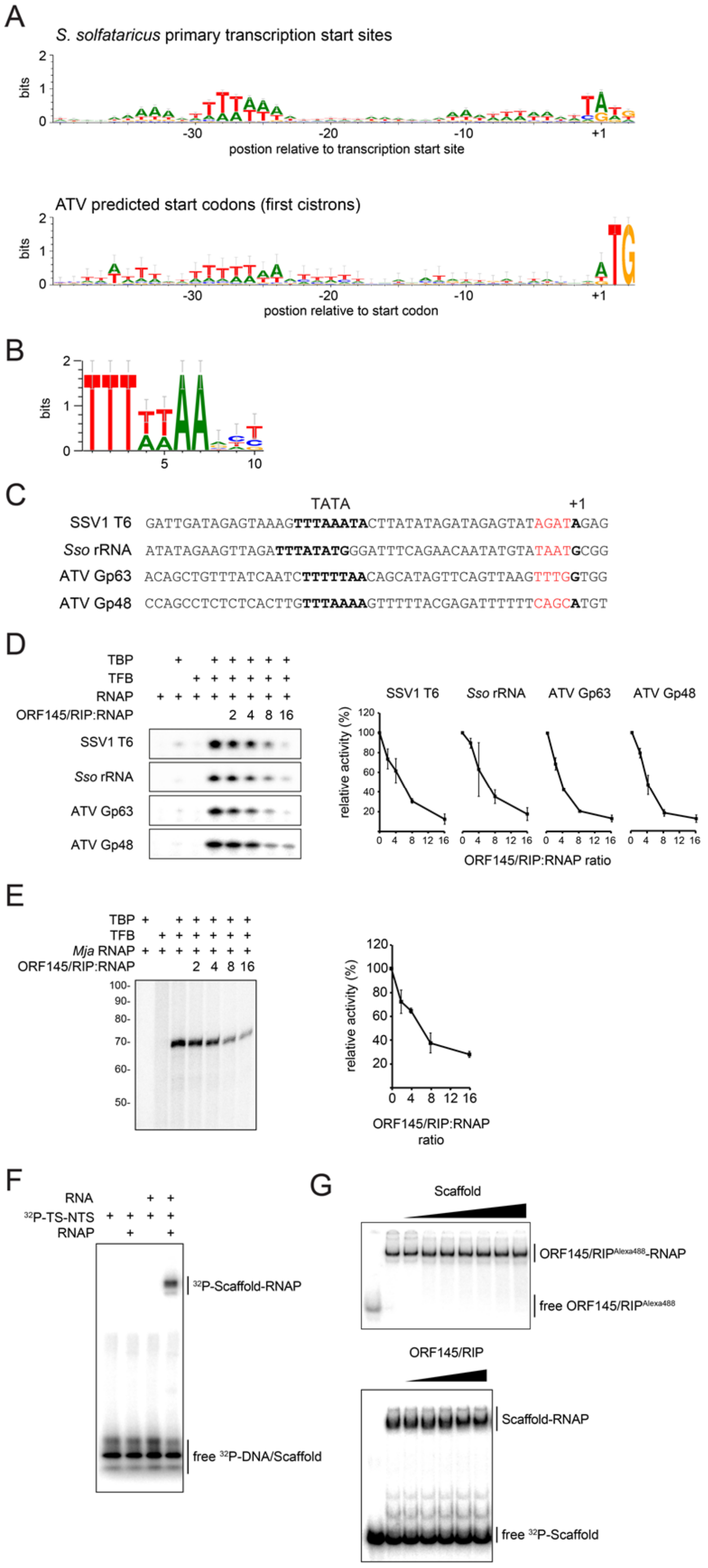
A
S. solfataricus primary transcription start sites
bits
postion relative to transcription start site
ATV predicted start codons (first cistrons)
postion relative to start codon
B
C
TATA
+1
SSV1 T6 GATTGATAGAGTAAAGTTTAAATACTTATATAGATAGAGTATAGATAGAG
Sso rRNA ATATAGAAGTTAGATTTATATGGGATTTCAGAACAATATGTATAATGCGG
ATV Gp63 ACAGCTGTTTATCAATCTTTTTAACAGCATAGTTCAGTTAAGTTTGGTGG
ATV Gp48 CCAGCCTCTCTCACTTGTTTAAAAGTTTTTACGAGATTTTTTCAGCATGT
D
TBP
TFB
RNAP
ORF145/RIP:RNAP
SSV1 T6
Sso rRNA
ATV Gp63
ATV Gp48
relative activity (%)
ORF145/RIP:RNAP ratio
E
Mja RNAP
F
RNA
32P-TS-NTS
32P-Scaffold-RNAP
free 32P-DNA/Scaffold
G
Scaffold
ORF145/RIPAlexa488-RNAP
free ORF145/RIPAlexa488
ORF145/RIP
Scaffold-RNAP
free 32P-Scaffold

22

**Supplementary Figure 4: ATV promoter prediction and additional transcription assays.**

(A) Sequence alignments of all 72 ORFs of ATV show that both TATA boxes and B-recognition elements are closely related to the Sso hosts promoters generated using WebLogo3 (http://weblogo.threeplusone.com/). (B) Putative consensus TATA element consensus (TTTWWAA) generated using MEME (http://meme-suite.org/index.html) on the ATV genome sequences -100 to +50 bps relative to the annotated translation start site (start codon), identified in 26 of the 46 predicted promoter regions with a distance of 25 ±5 bp from the start codon. (C) Non-template sequences for the SSV1 T6, Sso rRNA and ATV promoters used in the abortive transcription assays. The known and predicted TATA motifs are highlighted in bold and the non-complementary -4 to -1 regions are shown in red. (D) Abortive initiation assays of Sso and ATV promoters of the minimal PIC without TFE. (E) SSV1 T6 promoter-directed Mja transcription run-off assay in the absence of ORF145/RIP and presence of increasing ORF145/RIP to Mja RNAP molar ratios. A graphical representation of the % RNAP activity generated from 3 technical repeats is plotted as a function of ORF145/RIP:RNAP stoichiometry (right hand graphs on panel D and E). (F) EMSA showing that the formation of the transcription elongation complex (TEC) consisting of RNAP-DNA-RNA is strictly dependent on the RNA component. (G) Competition EMSAs showing that the DNA-RNA TEC nucleic scaffold does not compete with Alexa488 labeled ORF145/RIP for binding to RNAP, and reversely that ORF145/RIP does not compete with $^{32}$P-labeled-scaffold for RNAP binding.

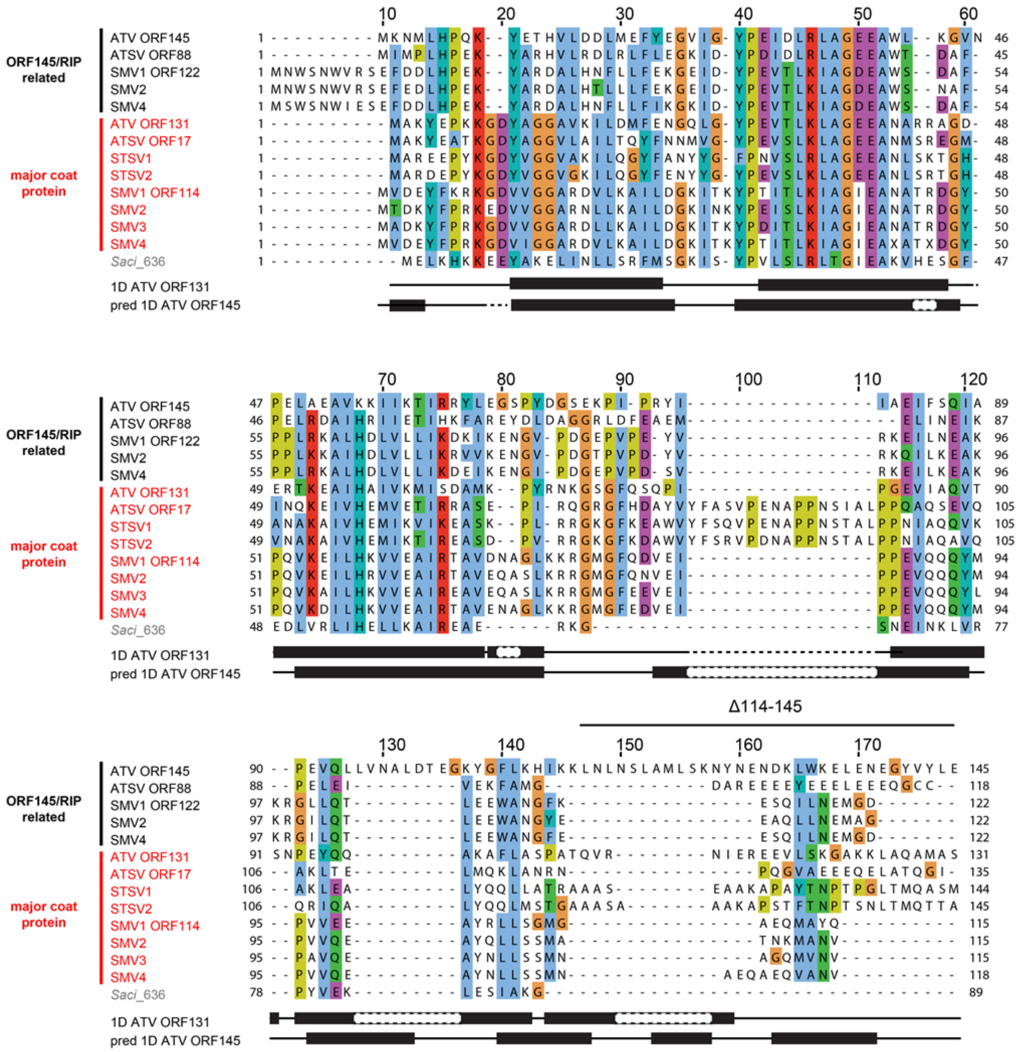


**Supplementary Figure 5: Sequence alignment of ORF145/RIP-like proteins.**

Sequence alignment of ATV ORF145/RIP and ORF131 and related proteins. Phylogenetic analysis (Figure 7B) suggests that the viral proteins fall into two different clades: ORF145/RIP-related and ORF131-related (major coat proteins). The secondary structure of ORF131 (pdb id: 3FAJ) and the predicted secondary structure of ORF145/RIP based on the I-TASSER homology model (Figure 6A) are depicted below the alignment with black boxes representing α-helices. Key to the sequences with gene identifier in brackets: ORF145/RIP related: ATV ORF145/RIP (75750440),

ATSV ORF88 [2], SMV1 ORF122 (589287071), SMV2 (730130337), SMV4 (933818242). Major coat proteins: ATV ORF131 (75750454), ATSV ORF17 [2], STSV1 (51980166), STSV2 (448260184), SMV1 ORF114 (589287076), SMV2 (730130344), SMV3 (933818150), SMV4 (933818251). An ORF131/ORF145/RIP related protein was also identified in *S. acidocaldarius*: *Saci*_0636 (499596793). The alignment was created using MUSCLE (http://www.ebi.ac.uk/Tools/msa/muscle/). It should be noted that an ORF145/RIP related protein was also identified in SMV3 (985760714), but due to the ambiguous sequence it was not included in the alignment. The residues that have been deleted in the truncation mutant ORF145$^{\Delta114\text{-}145}$ are indicated above the alignment.

**Supplementary Table 1: ORF145/RIP binding to RNAP does not restrict fluorescent dye mobility.**

| RNAP variant | Anisotropy* |
|---|---|
| $\text{RNAP}^{\text{Rpo1'-E257*DyLight550}}$ | 0.34 |
| $\text{RNAP}^{\text{Rpo1'-E257*DyLight550}}$ + ORF145/RIP | 0.32 |
| $\text{RNAP}^{\text{Rpo2''-Q373*DyLight650}}$ | 0.28 |
| $\text{RNAP}^{\text{Rpo2''-Q373*DyLight650}}$ + ORF145/RIP | 0.28 |

*Anisotropy values of fluorescent donor and acceptor dyes in single labeled RNAP and ORF145/RIP-RNAP complexes.

## Supplementary Table 2: List of oligonucleotides used in this study

| Oligonucleotide name | Sequence | Purpose |
|---|---|---|
| NdeI_ORF145_F | GGAATTCCATATGAAAAACATGCTCCACC | Cloning of ORF145 with thrombin cleavage site into pET21b+ |
| ORF145_thromb_XhoI_R | CCGCTCGAGGCTGCCGCGCGGCACCAGTTCTAAGTAC | Cloning of ORF145 with thrombin cleavage site into pET21b+ |
| ORF145_PKA__XhoI_R | CCGCTCGAGAACAGATGCACGACGTTCTAAGTAC | Cloning of ORF145 with protein kinase A recognition site into pET21b+ |
| ORF145_L114_XhoI_R | CCGCTCGAGGAGTTTTTTGATATGCTTCAGG | Cloning of ORF145 truncation mutant ORF145$^{\Delta 114\text{-}145}$ into pET21b+ |
| ORF145_A120C_F | CTCAATCTTAACTCATTATGCATGCTCTCAAAGAATTACAATGAG | Site directed mutagenesis of ORF145 to generate A120C substitution for Alexa488 labeling |
| ORF145_A120C_R | CTCATTGTAATTCTTTGAGAGCATGCATAATGAGTTAAGATTGAG | Site directed mutagenesis of ORF145 to generate A120C substitution for Alexa488 labeling |
| ORF145_NcoI_F | CATGCCATGGTGAAAAACATGCTCCACC | Cloning of ORF145 into pSVA1450 |
| ORF145_EagI_R | GGCCCGGCCGTCATTCTAAGTACACATAG | Cloning of ORF145 into pSVA1450 |
| ORF145_frameshift_Met5_F | CGTGATTAAGTTAACCATGGTGCAAAAACATGCTCC | Introduction of frameshift mutation after Met$^5$ |
| ORF145_frameshift_Met5_R | GGAGCATGTTTTTGCACCATGGTTAACTTAATCACG | Introduction of frameshift mutation after Met$^5$ |
| SSV1_T6_NTS | GATTGATAGAGTAAAGTTTAAATACTTATATAGATAGAGTATAGATAGAGGGTTCAAAAAATGGTT | EMSAs, abortive transcription, full length transcription |
| SSSV1_T6_TS | AACCATTTTTTGAACCCTCTATCTATACTCTATCTATATAAGTATTTAAACTTTACTCTATCAATC | Full length transcription |
| SSV1_T6_TS (-4/-1) | AACCATTTTTTGAACCCTCTGCTCATACTCTATCTATATAAGTATTTAAACTTTACTCTATCAATC | EMSAs, abortive transcription |
| Sso_rRNA_NTS | GGAATATATAGAAGTTAGATTTATATGGGATTTCAGAACAATATGTATAATGCGGATTCCCCCGCGGGAGAAACACTCCCGCCGGA | Full length transcription, abortive transcription |
| Sso_rRNA_TS | CCGGCGGGAGTGTTTCTCCCGCGGGGGAATCCGCATTATACATATTGTTCTGAAATCCCATATAAATCTAACTTCTATATATTCCA | Full length transcription |
| Sso_rRNA_TS (-4/-1) | CCGGCGGGAGTGTTTCTCCCGCGGGGGAATCCGCGCCGTACATATTGTTCTGAAATCCCATATAAATCTAACTTCTATATATTCCA | Abortive transcription |
| ATV_Gp63_NTS | AGGACAGCTGTTTATCAATCTTTTTAACAGCATAGTTCAGTTAAGTTTGGTGGCTCAGGAAATGGC | Abortive transcription |
| ATV_Gp63_TS (-4/-1) | GCCATTTCCTGAGCCACCAAGTAAAACTGAACTATGCTGTTAAAAAGATTGATAAACAGCTGTCCT | Abortive transcription |
| ATV_Gp48_NTS | CCAGCCTCTCTCACTTGTTTAAAAGTTTTTACGAGATTTTTTCAGCATGTCCAGCAATTATGTGCC | Abortive transcription |
| ATV_Gp48_TS (-4/-1) | GGCACATAATTGCTGGACATATCAAAAAAATCTCGTAAAAACTTTTAAACAAGTGAGAGAGGCTGG | Abortive transcription |
| Scaffold_NTS | CCGGCAGTACTAGTAATGACCAGGCGTAACTACTTCATCTTAACTACTCTAATGGATCTCCCATATGGTGGAGGTAAGGGTGG | Transcription elongation, EMSAs |
| Scaffold_TS | CCACCCTTACCTCCACCATATGGGAGATCCATTAGAGTAGTTAAGATGAAGTAGTTACGCCTGCTCATTACTAGTACTGCCGG | Transcription elongation, EMSAs |
| Scaffold_RNA | AUUUAGACCAGGCG | Transcription elongation, EMSAs |

## SUPPLEMENTARY REFERENCES


1 Combe, C. W., Fischer, L. & Rappsilber, J. xiNET: cross-link network maps with residue resolution. *Mol Cell Proteomics* **14**, 1137-1147, doi:10.1074/mcp.O114.042259 (2015).

2 Hochstein, R., Bollschweiler, D., Engelhardt, H., Lawrence, C. M. & Young, M. Large Tailed Spindle Viruses of Archaea: a New Way of Doing Viral Business. *J Virol* **89**, 9146-9149, doi:10.1128/JVI.00612-15 (2015).
